# Cold- and light-induced changes in the transcriptome of wheat leading to phase transition from vegetative to reproductive growth

**DOI:** 10.1186/1471-2229-9-55

**Published:** 2009-05-11

**Authors:** Mark O Winfield, Chungui Lu, Ian D Wilson, Jane A Coghill, Keith J Edwards

**Affiliations:** 1 School of Biological Sciences, University of Bristol, Bristol, BS8 1UG, UK; 2 School of Science and Technology, Nottingham Trent University, Nottingham, UK; 3 School of Life Sciences, University of the West of England, Frenchay Campus, Coldharbour Lane, Bristol, BS16 1QY, UK

## Abstract

**Background:**

For plants to flower at the appropriate time, they must be able to perceive and respond to various internal and external cues. Wheat is generally a long-day plant that will go through phase transition from vegetative to floral growth as days are lengthening in spring and early summer. In addition to this response to day-length, wheat cultivars may be classified as either winter or spring varieties depending on whether they require to be exposed to an extended period of cold in order to become competent to flower. Using a growth regime to mimic the conditions that occur during a typical winter in Britain, and a microarray approach to determine changes in gene expression over time, we have surveyed the genes of the major pathways involved in floral transition. We have paid particular attention to wheat orthologues and functional equivalents of genes involved in the phase transition in *Arabidopsis*. We also surveyed all the MADS-box genes that could be identified as such on the Affymetrix genechip wheat genome array.

**Results:**

We observed novel responses of several genes thought to be of major importance in vernalisation-induced phase transition, and identified several MADS-box genes that might play an important role in the onset of flowering. In addition, we saw responses in genes of the Gibberellin pathway that would indicate that this pathway also has some role to play in phase transition.

**Conclusion:**

Phase transition in wheat is more complex than previously reported, and there is evidence that day-length has an influence on genes that were once thought to respond exclusively to an extended period of cold.

## Background

In plants, the timing of the change from vegetative to reproductive growth is critical for successful reproduction, and must occur when both internal and external conditions are appropriate. The environmental cues of day-length and temperature have a strong influence on flowering, and the ability to perceive and respond to these cues is controlled through the photoperiod and vernalisation pathways, respectively [1].

Wheat (*Triticum aestivum *L.) is normally a long-day plant, flowering in spring and early summer when days are lengthening [2]. Additionally, wheat cultivars can be broadly divided into two categories, winter or spring, according to whether they require an extended period of cold to become competent to flower. In winter varieties, change from vegetative to reproductive phase is promoted by exposure to low temperatures (3°C – 8°C) for 4–6 weeks. These varieties are planted in the autumn so that seedlings are exposed to the cold of winter and so become competent to flower. However, they only become committed to flower as days lengthen in the spring. In addition to these two external factors, different wheat varieties can be distinguished by the intrinsic rate at which they tend to pass from floral induction to heading. This tendency is referred to as earliness *per se *[3].

In *Arabidopsis*, the genetic factors underpinning phase change have been well-characterised [4-6]. Four major genetic pathways regulate this transition: the photoperiod and vernalisation pathways mediate responses to the environmental cues of light and cold, respectively, whilst the autonomous and gibberellin pathways are dependent on endogenous signals [7-10]. Unfortunately, in studying phase transition in cereals one cannot draw directly on the information gained from the study of *Arabidopsis *since orthologues can't always be found. For example, cereals do not possess an orthologue of the *Arabidopsis FLOWERING LOCUS C *(*AtFLC*) gene, an important repressor of flowering [11,12]. To confuse matters more, genes with similar sequence don't necessarily have the same function. None-the-less, comparative genetics may still provide a promising starting point for a search of candidate genes involved in phase transition in the cereals. This certainly seems to be the case for the photoperiod pathway where there is a remarkable degree of conservation of functional components between *Arabidopsis *and rice [13]. However, the vernalisation pathway may have evolved independently in dicots and monocots such that they use different genes to retard flowering until winter has passed [8]. Thus, although much is known about the genes involved in floral transition in *Arabidopsis*, only recently have candidates for the key regulators determining vernalisation requirement in cereals been identified [14,15].

In the temperate cereals barley and wheat, two genes, *VRN1 *and *VRN2 *(unrelated to the identically named genes in *Arabidopsis*), have been reported to be the key elements in the vernalisation pathway [15-17]. Several papers addressing the issue of vernalisation have considered the interaction of just these two genes. A case in point is the model presented by Yan et al. [15] in which *TaVRN1 *and *TaVRN2 *are presented, respectively, as a promoter and repressor of flowering (see Additional file 1, for a schematic representation of this). According to the model, *TaVRN1 *is constitutively expressed in spring wheats but in winter varieties is up-regulated as a consequence of vernalisation. Conversely, *TaVRN2 *is highly expressed in winter varieties, thus repressing flowering, but not in spring varieties. It has been hypothesised that in winter wheats, extended periods of cold bring about down-regulation of *TaVRN2 *and, as a consequence, the up-regulation of *TaVRN1 *and commitment to flowering.

Using Affymetrix Genechip Wheat Genome Arrays as the platform for our analysis, we were able to consider the broad-scale response of the transcriptome to the changes in temperature and light that occur during a simulated autumn to winter transition. In this paper, however, we principally focus our attention on the components of the vernalisation pathway. However, our experimental design was such that we have also been able to draw conclusions about the impact on phase transition of several components of other flowering pathways. Finally, using a microarray approach rather than a more targeted approach allowed us to observe the expression profiles of genes that, *a priori*, we would not have assayed, and have been able to identify potentially important new players in phase transition.

## Results and discussion

The growth conditions were established to broadly simulate those that would be experienced by plants sown in October in Britain. In particular, attention was paid to the details of suitable light quality and photoperiod (Table 1), because there is evidence that sustaining high light intensity and an extended photoperiod while reducing temperature may result in stress related patterns of gene expression [18].

**Table 1:** Growth condition for the time-course experiment (PAR = photosynthetically active radiation).

Duration (days)	Weeks after germination	Temperature (°C)		Day-length (h)	PAR
		Day	Night		
21	3	16	14	14	280
7	4	14	12	14	280
7	5	14	10	12	185
7	6	12	10	11	185
7	7	12	8	11	185
7	8	10	6	10	95
7	9	8	4	9	95
7	10	6	2	8	95
7	11	4	2	8	95
7	12	2	2	8	48

**Figure 1:**
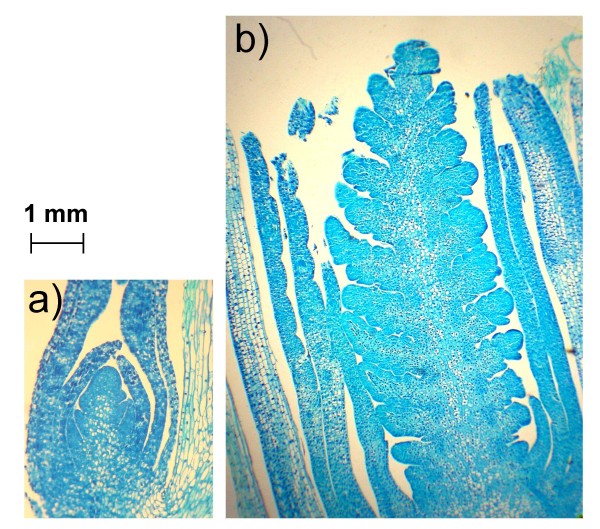
**Dissected crown tissue from 6 week old plants that have experienced a gradual decline in temperature and light: a) Solstice, a winter variety; b) Paragon, a spring variety**. The two images are at the same magnification.

Winter (Harnesk and Solstice) and spring wheat (Paragon) varieties of wheat were grown at 16°C day/14°C night for 21 days, and then, over a period of nine weeks, exposed to a slow, stepped decline in temperature and light (Table 1). The developmental state of the crown tissue was assessed in thin tissue sections at the end of each temperature stage (every 7 days from the third week onwards). As shown in Figure 1, after six weeks' growth, crowns in the spring variety were much more advanced in their development than those in the two winter varieties – while the apices of the winter varieties were at approximately stage 2 (elongation and early formation of leaf primordia), according to the scale proposed by Gardner et al. [19], in Paragon a distinct spike with enlarging spikelets and early glumes was evident (stage 5 or 6). Indeed, apices of Paragon showed signs of double ridge formation as early as the fifth week. The winter varieties were assessed to be fully "vernalised" 12 weeks post-germination, and plants treated in this way went on to flower when returned to warmer, long-day conditions (see Additional file 2). Unvernalised, plants of Harnesk and Solstice did not flower (see Additional file 2, A).

**Figure 2:**
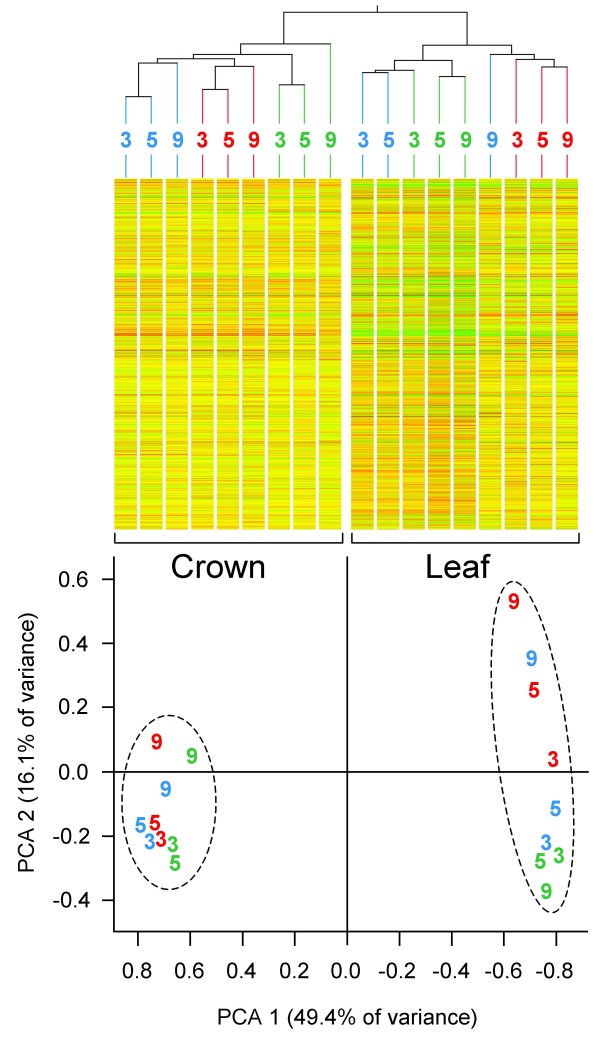
**Condition tree and PCA plot based on gene expression profiles of the three cultivars for 3 time points: 3 weeks, 5 weeks and 9 weeks post-germination (marked on plots)**. In both plots, the samples of Harnesk are highlighted in blue, Solstice in red, and Paragon in green.

Global gene expression profiles showed there to be differences between the three varieties, between the two tissues (crown and leaf) and across the time course of the experiment (GEO accession number for array data is GSE11774). The most marked difference in gene expression was between leaf and crown (Figure 2) with 22.8 – 28.4% of the transcripts being differentially expressed (Table 2). In contrast, pair-wise comparisons between the cultivars at the end of three weeks, at which time all plants had received exactly the same treatment, showed only 1.5 – 3.7% of transcripts in crown tissue and 1.3 – 2.8% in leaf tissue to be differentially expressed (Table 3).

**Table 2:** The number of statistically significant differences in gene expression between crown and leaf.

	Crown vs Leaf		
	
	Harnesk	Paragon	Solstice
2 Fold	17427	13977	14501
5 Fold	6129	5277	5394

Values derived from a volcano plot of 2 and 5 fold differences with t-test p value of 0.05.

**Table 3:** The number of statistically significant differences between cultivars three weeks post germination.

	Harnesk vs. Paragon		Harnesk vs. Solstice		Paragon vs. Solstice	
	
	Crown	Leaf	Crown	Leaf	Crown	Leaf
2 Fold	1326	775	920	1236	1943	1734
5 Fold	268	186	168	318	299	437

Values derived from a volcano plot of 2 and 5 fold differences with t-test p values of 0.05.

In both tissues, the transcripts of thousands of genes showed statistically significant changes in abundance across the time-course. However, these global patterns do not interest us in this paper. Instead, we focus on the dynamics of expression of genes involved in phase transition. That is, initially we consider the small number of genes reported to be specifically involved in vernalisation in wheat, and then briefly focus on reported orthologues and functional equivalents of the components of the other three pathways involved in phase transition in *Arabidopsis*. Finally, we consider all those features annotated as MADS-box genes.

### Vernalisation in wheat

**Figure 3:**
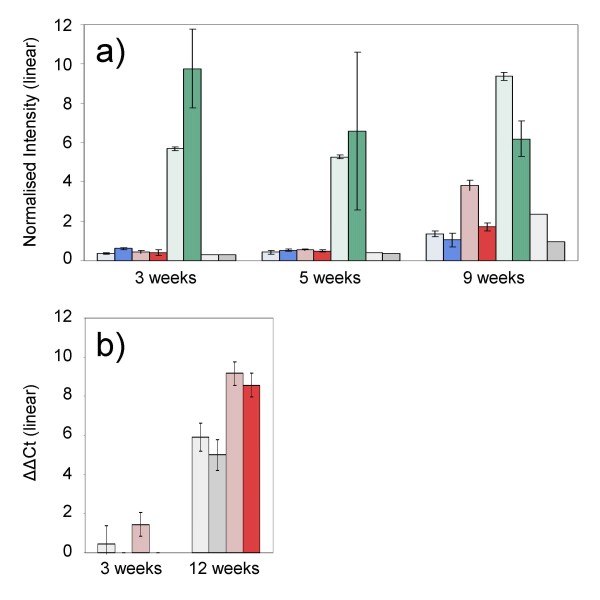
**Bar diagram of *TaVRN1 *transcript abundance: a) a comparison of array data of the three varieties at 3, 5 and 9 weeks; b) a comparison of qRT-PCR data of vernalised Solstice and non-vernalised controls at 3 and 12 weeks**. Colour code: Harnesk = blue; Solstice = red; Paragon = green; Control = grey; in each case the lighter shade represents crown tissue.

In our study, the profiles of transcript abundance of *TaVRN1*, the proposed major promoter of flowering, were entirely consistent with those reported in other studies [15,20,21]. At three weeks, prior to exposure to cold, *TaVRN1 *transcript was much more abundant in Paragon than in the two winter varieties (Figure 3a). In Paragon, transcript levels remained high across the time-course. In the winter varieties, transcript levels for *TaVRN1 *were initially low but by 9 weeks had increased, and by 12 weeks, when plants were assessed to be fully vernalised (plants transferred to long days at 16°C went on to flower – see Additional file 2, B), there had been an approximately 10 fold increase in abundance. These patterns, confirmed by qRT-PCR (Pearson Correlation Coefficient = 0.76), are consistent with the hypothesis that *TaVRN1 *is a promoter of flowering induced by extended periods of cold. However, our experimental design gave us the opportunity to make an additional and interesting observation with regard the regulation of *TaVRN1*. That is, the *TaVRN1 *transcript accumulated in a similar fashion, although to a lesser extent, in both vernalised plants and controls – plants exposed to a gradual decline in light intensity and day-length but not a decline in temperature (Figures 3a, b). Our results suggest that *TaVRN1 *expression might be influenced by both light and temperature, as has recently been reported by Hemming *et al*. (2008). Indeed, the two stimuli might act synergistically since there was greater accumulation of transcript in the vernalised plants, which experienced the influence of both cues, than in the control plants, which experienced only a decline in day-length. Alternatively, we cannot exclude the possibility that this important promoter of flowering gradually accumulates through time allowing plants to eventually become competent to flower even in the absence of cold. Thus, given the later hypothesis, even following a mild winter, winter varieties would eventually accumulate enough TaVrn1.p to permit flowering.

Whereas, *TaVRN1 *behaved in a manner consistent with it being a promoter of flowering, the profiles of abundance of the *TaVRN2 *transcript were not what would be expected of a repressor of flowering, and do not fit the model presented in Additional file 1 (model a). In crown tissue, where one might have expected to see a response, since the perception of cold is thought to occur in actively dividing cells of the shoot apical mersitem, transcript abundance remained low (below the level of detection with qRT-PCR) and unchanging in all three varieties. In leaves of the winter varieties, there was a decline in abundance between weeks five and nine – a 2.5-fold and 1.7-fold decline, respectively, in Harnesk and Solstice. But, even at their highest point, transcript levels were very low – result confirmed by qRT-PCR (Pearson correlation = 0.95). The lack of correspondence between our results for *TaVRN2 *and those of other groups may find explanation in the different experimental designs used. Our growth conditions were designed to mimic the autumn to winter transition. In other studies, continuous long-day condition were used in combination with either constant low temperature (4°C), or a one-phase shift from high to low temperature [2,16,21]. Regardless of the differences in growth conditions, the precise role of Ta*VRN2 *in vernalisation has already been questioned. In a recent paper, [22] it was shown that its expression remained low when plants were vernalised under short day conditions, and suggested that *TaVRN2 *is probably not a repressor of *TaVRN1*. In addition, it has been suggested that TaVrn2.p is not able to bind the *TaVRN1 *promoter [12] and so can't act as a direct repressor of its expression. Our results for *TaVRN2 *correspond with those of Trevaskis *et al*. [22] then, rather than with results that indicate a direct role for *VRN2 *in vernalisation.

Recent discussions of the genetic control of phase transition in barley and wheat have not only brought into question the role of *TaVRN2 *[23,24] but indicate that genes once thought to be exclusively controlled by temperature may also respond to day-length [2,12,25,26]. A third gene, *TaVRN3*, a component of the photoperiod pathway thought to be a promoter of *TaVRN1 *expression, is up-regulated by long-days. Alternative models for the relationship between these three genes are depicted in Additional file 1 (models a and b). The third component of the models of phase transition presented in Additional File 1 is *TaVRN3*, which is thought to be an orthologue of *AtFT *(*FLOWERING LOCUS T*). According to Yan and co-workers [24], TaVrn3.p is a flowering promoter which is up-regulated by long days. In turn, TaVrn3.p positively regulates *TaVRN1*. In this model, both *TaVRN1 *and *TaVRN3 *are negatively regulated by TaVrn2.p. Vernalisation results in a decrease in TaVrn2.p and the de-repression of *TaVRN1 *and *TaVRN3*. Significantly, it was suggested [24] that transcript levels of all three genes remain low under short day conditions, and that only on transfer to long days are *TaVRN1 *and *TaVRN3 *up-regulated and able to initiate the cascade of events that result in flowering. Our results for *TaVRN3 *agree with those of Yan *et al*. [24]: transcript levels of *TaVRN3 *remained low and unchanging throughout the experiment in all three varieties. That is, the plants were grown under short-day conditions, and so *TaVRN3*, which requires long-days, was not expressed.

This negative result is, of itself, quite interesting since it indicates that TaVrn3.p is not required for *TaVRN1 *to be expressed; regardless of the low abundance of the *TaVRN3 *transcript, transcripts of *TaVRN1 *exhibited an increase over the time-course.

In the light of the evidence against *TaVRN2 *being a repressor of *TaVRN1*, the group of Trevaskis [23,27] has recently proposed a model in which *TaVRN2 *is presented as a possible integrator of the vernalisation and photoperiod pathways. In this model, the principle relationship between *TaVRN1 *and *TaVRN2 *has been reversed with respect to earlier models; that is, *TaVRN1 *negatively regulates *TaVRN2 *(see Additional file 1, model b). In this model, *TaVRN2 *is up-regulated under long-days before the onset of winter and, in the absence of TaVrn1.p, represses *TaVRN3*. After vernalisation (after winter, therefore), even though days are lengthening, TaVrn1.p is abundant and represses *TaVRN2*, which, in turn, removes the repression of *TaVRN3 *(synonymous with *TaFT1*). This model might go some way to explain the two steps of phase transition: i) competence to flower as a result of the cold of winter; ii) commitment to flowering as temperatures rise and days lengthen in the spring.

### Wheat orthologues and functional equivalents of Arabidopsis floral pathway genes

There are four major genetic pathways that regulate vegetative to reproductive transition in *Arabidopsis*: the photoperiod and vernalisation pathways which mediate responses to light and cold, respectively, and the autonomous and gibberellin pathways that are regulated by endogenous signals [7-10]. We tried to identify all the probe-sets on the Affymetrix Genechip Wheat Genome Array that correspond to orthologues, or are functional equivalents, of *Arabidopsis *genes involved in the four pathways that control flowering (see Additional file 3). Obviously, this was not possible in all cases. However, the major components of the photoperiod pathway in *Arabidopsis *do have orthologues in the cereal monocots [8,28,29], and two of the major component of the autonomous pathway in *Arabidopsis*, *AtFCA *and *AtFY*, are also conserved in monocots [28]. Using both nucleotide and protein sequences of *Arabidopsis *genes, BLAST searches were made to identify cereal genes with high sequence similarity. These sequences were then used to search the NETAFFX database of probe-sets on the Wheat Array . Of the 24 targets that correspond to *Arabidopsis *phase transition genes, nine didn't show statistically significant changes in abundance (two-fold, p 0.05), and were not considered further. The other 15 showed abundance profiles that included two-fold or greater change.across the time-course.

### GA pathway genes

**Figure 4:**
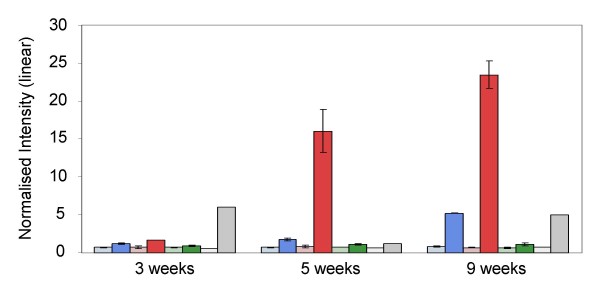
**Bar diagram of Ent-kaurene oxidase transcript abundance: a comparison of array data (normalised intensity) of the three varieties at 3, 5 and 9 weeks**. Colour code: Harnesk = blue; Solstice = red; Paragon = green; Control = grey; in each case the lighter shade represents crown tissue.

The Affymetrix array doesn't include probe-sets for *AtGA1 *(codes for ent-copalyl diphosphate synthase) or *AtGA INSENSITIVE *(the wheat orthologue is *REDUCED HEIGHT B1 *[*RHT B1*]). There is a probe-set for *AtRGA1 *(the wheat orthologue of *RHT D1*), but we did not observe differential accumulation of this gene. However, there was a clear genotype-dependent, cold response of some components of the gibberellin pathway: transcripts for *ent*-kaurene synthase and *ent*-kaurene oxidase (correspond to *AtGA2 *and *AtGA3*, respectively), showed leaf specific accumulation (> 20-fold increase after 12 weeks) in the two winter varieties and no response at all in Paragon (Figure 4). This result was confirmed by qRT-PCR (Pearson correlation = 0.99). *Ent*-kaurene synthase and *ent*-kaurene oxidase are two of the principal enzymes of the gibberellin biosynthetic pathway [30,31]. Thus, given the profiles of abundance that we observed, one might assume that, in the two winter varieties, there was an increase in gibberellins. In Arabidopsis, gibberellic acid (GA) activates the expression of *AtSOC1*(*SUPPRESSOR OF OVER EXPRESSION OF CO 1*) [32], an important integrator of several flowering pathways (discussed below), which in turn promotes flowering through its action on floral meristem identity genes or their products (Komeda 2004). What's more, Moon *et al*. [32] report that the gibberellin pathway is the only pathway to promote flowering under short days. Thus, it would appear that we have evidence to show that in wheat the gibberellin pathway functions in a similar manner to that in *Arabidopsis*, and that as a consequence of vernalisation under short-days it tends to promote flowering. However, the complete lack of response in the spring variety, Paragon, is intriguing: does the gibberellin pathway not function to promote flowering in spring varieties of wheat?

### Autonomous pathway

In Arabidopsis, seven genes (*AtFCA*, *AtFY*, *AtFLD*, *AtFVE*, *AtFPA*, *AtLD *and *AtFRI*) have been shown to comprise the autonomous pathway [7,8]. All of these, except *AtFRI*, are thought to promote flowering by repressing *AtFLC*, a dominant repressor of flowering. *AtFRI*, on the other hand, upregulates expression of *AtFLC *and so represses flowering. No orthologues for *AtFRI *or *AtFLC *have been found in the cereals (Alexandre and Hennig, 2008). Orthologues (or, at least, genes which code for proteins with similar structure and function) of the other six are present in the cereals [28], but there are probe-sets for only two of these (*TaFVE *and *TaFCA*) on the wheat array (see Additional file 3). Both these genes showed a slight increase in abundance across the time-course. However, there were no particular differences in response between the winter and spring varieties. In addition, in vernalised and control plants transcripts for the two genes exhibited similar profiles of abundance, as might be expected for genes that are not thought to be responsive to light or cold [8].

### Photoperiod pathway

The principal components of the photoperiod pathway are conserved in the monocots and, more pertinent to this discussion, in the cereals [8,33,34]. On the Wheat Genome Array, there are probe-sets that correspond to many of the genes belonging to the photoperiod pathway (Additional file 3).

In *Arabidopsis*, *AtCONSTANS *(*AtCO*) encodes a transcription factor that activates genes required for floral initiation. It integrates circadian clock and day-length signals and, under long-days, activates the floral promoters *AtFT*, *AtSOC1 *and *AtLFY *[8]. The two circadian clock genes *AtLHY *and *AtTOC1 *influence the expression of *AtCO*. They form part of a feedback mechanism, each directly affecting the expression of the other: AtLhy.p is a repressor of *AtTOC1*, and AtToc1.p is required for the expression of *AtLHY *[8]. The cyclic expression of these two genes, which occurs over a 24 hour period, entrains that of *AtGIGANTEA *(*AtGI*). This latter activates *AtCONSTANS*.

In this study, the profiles of abundance for *TaLHY *and *TaTOC1 *were complementary to each other, as one might expect from their relationship to each other in circadian cycling. In crown tissue, transcript of *TaLHY *increased in abundance and then declined; conversely, that of *TaTOC1 *declined and later increased. The profile for *TaGI *was very similar to that of *TaTOC1 *(see Additional file 4). Given that in both rice and *Arabidopsis*, *GI *is a promoter of *CO *expression [8], one might have expected the transcript for *HEADING DATE 1 *(*TaHD1*), the supposed wheat orthologue of *AtCONSTANS*, to follow the profile of *TaGI*. However, it did not show differential expression in this study. Interestingly, other transcripts that appear to be members of the *CONSTANS-like *family of genes exhibited profiles that did reflect those of *TaLHY*, and *TaTOC1*, and *TaGI*. In particular, a sequence highly similar to barley *CONSTANS-like *9 (the most divergent of the barley *CONSTANS-like *genes which has no counterpart in *Arabidopsis *[35]) had a profile of abundance very similar to that of *TaLHY *(see Additional file 4). A transcript with similarity to *CONSTANS-like 1 *in *Lolium perenne*, a gene which has been reported to increase after extended periods of exposure to cold [36], had a profile of transcript abundance that echoed that of *TaTOC1 *and *TaGI*. Ciannamea *et al*., using a similar experimental approach to that used in this study, suggested that the profile of transcript abundance for *LpCOL1 *was suggestive of the gene being involved in the vernalisation response [36]. We observed a very similar profile of responses in both the cold treated plants and the controls. This would suggest that this gene in wheat is responding to shortening day length.

The phytochromes (perceive red and far-red light) and the cryptochromes (perceive blue and UV-A light) are the principle components involved in the perception of light. What is more, phytochromes regulate a variety of developmental processes, and are thought to be involved in signaling, probably through the possession of a kinase domain residing within their C-terminal domain [13]. Within the photoperiod pathway, it is believed that, under long-day conditions, these photoreceptors contribute to the initiation of flowering through the stabilisation of the CO protein [37]. Of the principal photoreceptors, only three, *TaCRY2*, *TaPHYA *and *TaPHYC*, were identified on the Affymetrix Wheat Genome Array. Two of these, *TaCRY2 *and *TaPHYA*, exhibited a response under our experimental conditions. Transcript of *TaCRY2 *accumulated, principally in leaf tissue, of the two winter varieties under both the vernalisation regime and in the control plants. In Paragon, no statistically significant change in transcript abundance was observed. Transcript levels of *TaPHYA *were initially much higher in the crown tissue of the two winter varieties than in Paragon and increased across the time course. There was also an increase in transcript abundance in leaf tissue of the winter varieties, but from a lower initial level. The same pattern of increase was seen in the controls.

### Intergrative pathway

**Figure 5:**
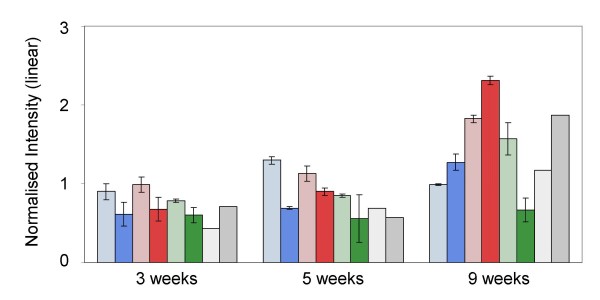
**Bar diagrams of transcript abundance of the MADS-box gene *TaAGL21*, the gene most similar to *AtSOC1*: a comparison of array data (normalised intensity) of the three varieties at 3, 5 and 9 weeks post-germination**. Colour code: Harnesk = blue; Solstice = red; Paragon = green; Control = grey; in each case the lighter shade represents crown tissue.

In *Arabidopsis*, the four floral pathways converge through genes of the integrative pathway [7]. The activation of floral integrators, such as *AtFLOWERING LOCUS T *(*AtFT*) and *AtSUPPRESSOR OF CONSTANS 1 *(*AtSOC1*), in turn, lead to the activation of floral meristem identity genes such as *LEAFY *(*LFY*) and *APETALA1 *(*AP1*). We did not observe differential expression of *TaFT *(see above discussion of *TaVRN3*). On the other hand, transcripts for wheat genes that share sequence similarity with *AtSOC1 *did show differential expression. Zhao *et al*. [38] identified seven MADS-box genes (*TaAGL1*, *TaAGL7*, *TaAGL18*, *TaAGL20*, *TaAGL21*, *TaAGL23 *and *TaAGL38*) that, upon phylogenetic analysis, were placed in the SOC1-like clade of MADS-box genes. Only three probe-sets on the wheat array corresponded with these seven genes (see Additional file 5). The gene *TaAGL7 *corresponds with the probe set Ta.25343. The genes *TaAGL1*, *TaAGL18 *and *TaAGL23 *(the most similar to *AtSOC1*) all correspond to one probe-set (Ta.21250), and *TaAGL20*, *TaAGL21 *and *TaAGL38 *(the least similar to *AtSOC1*) to another (TaAffx.19661) – given the high sequence similarity between the genes in the respective groups, they are probably homoeologues and so we cannot report on their individual behaviour. However, the three probe-sets that correspond to the genes of the SOC1 clade of MADS-box genes all evidenced essentially the same profile of abundance (data not shown). That is, in both leaf and crown tissue of all three varieties, transcript increased slightly (Figure 5).

In *Arabidopsis*, *AtSOC1 *acts as a integrator of several floral induction pathways, and is induced by vernalisation [39]. Genes from both rice (*OsMADS50*) and ryegrass (*LpMADS1*, *LpMADS2*, *LpMADS3*) that share sequence similarity with *AtSOC1*, and may be functional equivalents of it, are also reported to accumulate as a consequence of vernalisation [40,41]. We observed a slight increase in abundance in wheat, but the profile of abundance in the vernalised and control plants were essentially identical. Thus, we might not be observing a response to declining temperature but to declining day-length. Alternatively, as indicated by Shitsukawa *et al*. [42], it might well be that this gene is neither influenced by vernalisation nor day-length. Indeed, this latter group present a model in which *WSOC1 *acts as an activator of flowering that is influenced by the Gibberellin pathway [42].

### Other MADS-box genes

Members of the MADS-box gene family encode transcription factors that play a fundamental role in signal transduction and control of development in probably all eukaryotes [43]. For instance, floral organ identity genes (homeotic genes) of the ABCDE model of floral organ development are mostly MADS genes [44]. In rice and *Arabidopsis*, species for which sequencing has been completed, 73 and 107 MADS-box genes have been identified, respectively [38]. In wheat about 50 MADS-box genes have been identified; these are dispersed throughout the genome [38,45]. We identified all the features on the wheat array that correspond with MADS-box genes (see Additional file 5) to determine whether they exhibited abundance profiles indicative of involvement in phase transition.

The MADS-box gene *VEGETATIVE TO REPRODUCTIVE TRANSITION 2 *(*TaVRT2*), the product of which shares 51% sequence identity with the *Arabidopsis *protein SHORT VEGETATIVE PHASE (AtSvp.p), has been reported to play an important role in phase transition in wheat [12,46]. Kane *et al*. [46] reported that, in spring wheat, levels of TaVrt2.p remain low and stable under cold treatment, whilst in winter wheat it starts higher than in spring wheat and then declines. This pattern of expression, reminiscent of that reported for *TaVRN2*, is consistent with the hypothesis that *TaVRT2 *is a repressor of flowering. Indeed, Kane *et al*. [12] suggested that TaVrt2.p (as part of a hypothesised protein complex, possibly with TaVrn2.p) might bind to the promoter region of *TaVRN1 *and repress it. However, Trevaskis *et al*. [12,26], working with barley, suggested that this is unlikely to be the case: they failed to find any evidence for there being a direct interaction between *HvVRT2 *and *HvVRN1 *(the barley homologues of *TaVRT2 *and *TaVRN1*, respectively) and showed that *HvVRT2 *transcript abundance increased during cold treatment. In our study, in both tissues of all three varieties, there was an increase in transcript abundance (approx. 1.5 to 2.0 fold) as temperature decreased. Thus, our results are in agreement with those of Trevaskis *et al*. [26]. If indeed *VRT2 *is a repressor of flowering, the profiles observed here could be explained by assuming that, in both winter and spring varieties, there is a tendency to retard flowering until permissive warm, long-day conditions return. That is, *TaVRT2 *might not repress *TaVRN1 *but counteract its function and so be part of a mechanism to hold back flowering until the return of spring. Indeed, it has been reported that short days repress reproductive development in spring varieties [47]. That is, plants that have no vernalisation requirement use day-length as a cue to retard flowering until the permissive temperatures and lengthening days of spring stimulate them to flower. Thus, *TaVRT2*, a repressor of flowering, and *TaVRN3*, a floral promoter, might work in concert as part of a mechanism to check the flowering in vernalisation saturated plants (or, indeed, in plants that don't require cold to acquire competence to flower) until day-length is appropriate.

Finally, several less well studied MADS-box genes behaved in an intriguing manner suggestive of their involvement in vernalisation or photoperiod induced phase transition. Of particular interest were *TaAGL10*, *TaAGL33 *and *TaAGL42*. The gene *TaAGL10 *belongs to the same subfamily of MADS-box genes as *TaVRN1 *although their proteins shares only 52% identity [38]. Interestingly their profiles of expression in crown tissue were quite similar – there was little response of *TaAGL10 *in leaf tissue of any of the three varieties. That is, at all three time points, transcript in Paragon was much higher than in the two winter varieties. In the latter, transcript was initially very low but began to accumulate by the ninth week. Analysis with qRT-PCR showed that this increase continued to the twelfth week (data not shown). The similarity between the profiles of *TaVRN1 *and *TaAGL10 *suggests that they might respond to the same cues. Further work will need to be carried out to determine the downstream interaction of the TaAgl10 protein.

**Figure 6:**
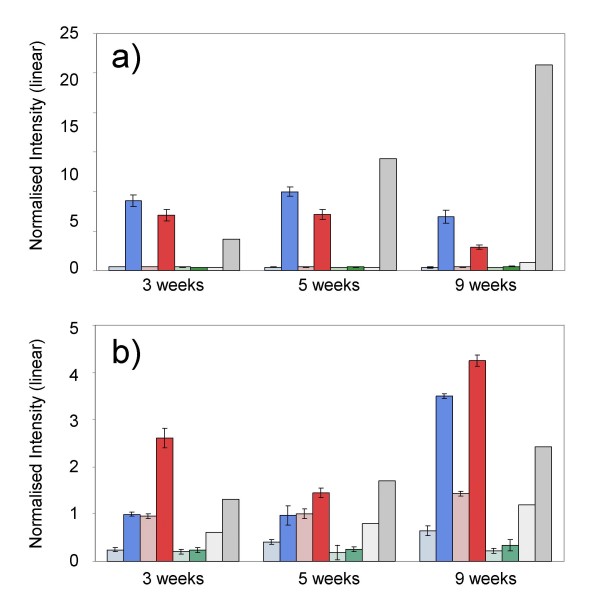
**Bar diagrams of transcript abundance of a) *TaAGL33 *and b) *TaAGL42*: a comparison of array data (normalised intensity) of the three varieties at 3, 5 and 9 weeks post-germination**. Colour code: Harnesk = blue; Solstice = red; Paragon = green; Control = grey; in each case the lighter shade represents crown tissue.

**Figure 7:**
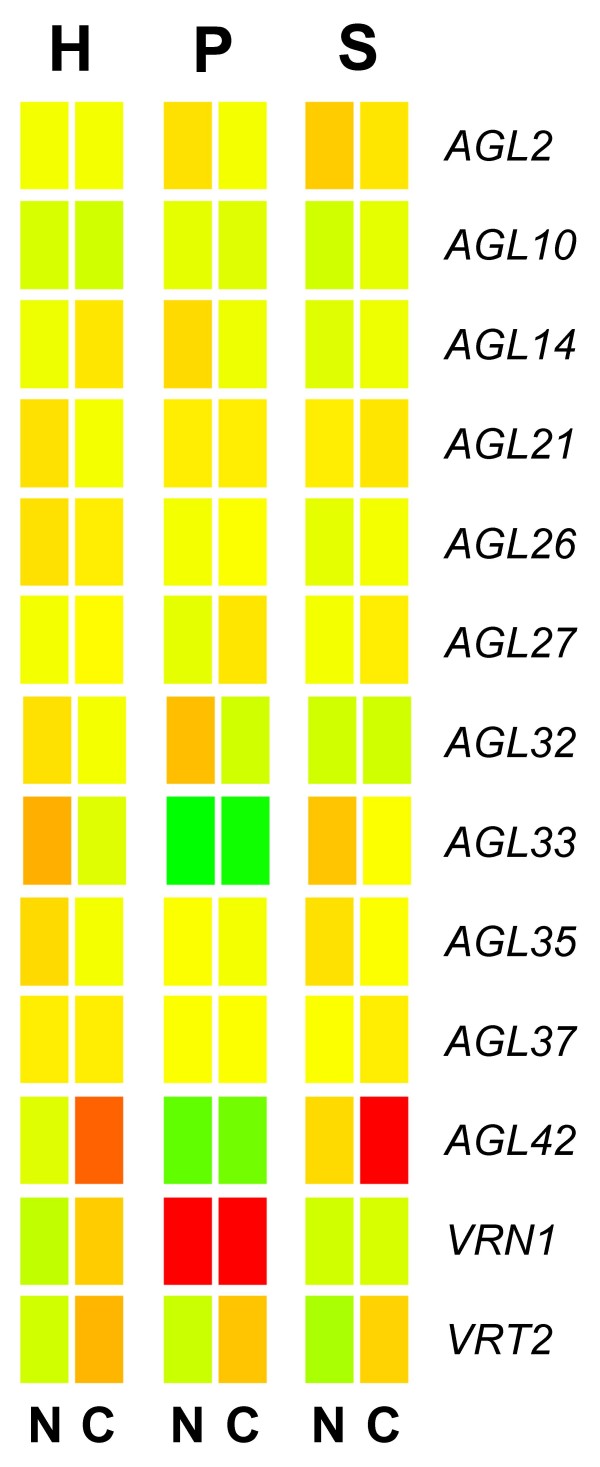
**Relative expression of MADS-box genes in plants exposed to a sudden drop in temperature from 15°C to 4°C**. Most MADS-box genes didn't show a statistically significant response to the cold shock. In the two winter varieties, *TaAGL42 *showed a marked increase in expression in the cold treated plants relative to the controls (> 3-fold change, *p *= 0.05).*TaVRT2 *showed a >1.5-fold increase in all three varieties (*p *= 0.05). Code: H = Harnesk; P = Paragon; S = Solstice; N = no cold treatment; C = cold shock.

The two genes *TaAGL33 *and *TaAGL42 *are Class I MADS-box genes. The precise function of genes belonging to this class is not well understood [48,49]. However, both *TaAGL33 *and *TaAGL42 *are closely related to the rice gene *OsMADS51*[38] which, in rice, has been shown to be a flowering activator under short-days[50]. In our experiment, differential expression of these two genes was restricted to the two winter varieties (Figure 6). At three weeks, transcript of *TaAGL33 *was relatively high in leaf tissue of the two winter varieties, but declined between the fifth and ninth weeks. This pattern corresponds with the results reported by Trevaskis *et al*., [21] for the Type I MADS-box gene, *TaMx23*. A BLAST search using the sequence of TaMX23 (accession number BJ258117) shows that the two sequences are very similar (85% identify). However, *TaMx23 *is more similar to the sequence of *TaAGL42 *(95% identity) which showed a different profile of abundance. Transcript of *TaAGL42 *increased, principally in leaf tissue, in the winter varieties and showed no response in Paragon. If these two MADS-box genes are involved in cold induced phase transition, their profiles of abundance would suggests that *TaAGL33 *is a repressor of flowering and *TaAGL42 *a promoter. The complete lack on response of both genes in the spring wheat, Paragon, is quite intriguing; it would indicate that in spring varieties these genes are constitutively repressed or that they occur as non-functional alleles. This would make sense for the *TaAGL33 *transcript assuming that it were a repressor of flowering. That is, in winter varieties it declines as a consequence of vernalisation, whilst in spring varieties it always remains low or absent. However, the role that *TaAGL42 *might have in phase change is more difficult to interpret. If one were to hypothesise that *TaAGL42 *generally acts to promote flowering then it should be constitutively expressed in spring varieties: it remained low and unchanging in both tissues, however. Thus, *TaAGL42 *might not be involved in phase transition at all. A clue that instead it might be involved in cold acclimation is given by the fact that, of the MADS-box genes on the array, it is the only one apart from *TaVRT-2 *that responded to a cold "shock". That is, in a separate experiment (not described here) in which plant were exposed to a rapid drop in temperature from 15°C to 4°C and held for two days, transcript of *TaAGL42 *in the two winter wheat varieties increased in abundance four- to five-fold over a two day period. Transcript for *TaVRT-2 *approximately double in all three varieties over the same period whilst transcript for all other MADS-box genes studied showed no statistically significant change in abundance (Figure 7).

## Conclusion

This study, we believe, is novel in that it was carried-out under conditions that closely reflect those that typically occur during the winter in a northern climate – a gradual decline in temperature, day-length and light intensity. In most previous studies, unnaturally high light intensities have been maintained throughout the experiment, or sudden drops in temperature have been used. These conditions may have given rise to unusual, stress-related responses. Certainly, we have observed expression patterns for some genes that call into question earlier studies.

In choosing a microarray approach we have obtained a broad overview of the behaviour of the wheat transcriptome to winter conditions and this has provided many interesting clues to the interaction of the several pathways involved in phase transition. We have also seen how these might differ between different tissue and, particularly, between winter and spring varieties of wheat. With regard to many genes, we observed patterns of transcript accumulation that reflect those seen by other groups in other studies. However, as a consequence of the conditions under which we grew our plants, we have also made observations that give additional insight to the complexities of the interaction that occur between floral pathways, and how internal and external cues come into play to ensure flowering occurs when internal and external cues are appropriate. For instance, it has not been previously reported that *TaVRN1 *might respond to light – thus, it might be that *TaVRN1 *is involved in pathways other than just the vernalisation pathway. The lack of a clear response of Ta*VRN2 *was also unexpected when we began our study, but has recently been discussed by other groups [22]. Our results support the view that this gene is probably not directly involved in vernalisation as was, until recently, thought to be the case. Clear evidence that the Gibberellin pathway is important in wheat and, by extension, in other cereals too, is also interesting. Finally, we have highlighted genes that certainly warrant further investigation such as the MADS-box genes *TaAGL10*, *TaAGL33 *and *TaAGL 42*.

On a broad scale, it seems evident that phase transition and flowering in cereals, as in *Arabidopsis*, is controlled by several different pathways that allow plants to respond to different external (temperature, day length and light quality) and internal (gibberellin and autonomous pathways) cues. The different pathways, each responding to their own cues and each promoting or repressing flowering depending on the message provided by these cues, provide the checks and bounds to flowering. Thus, only when plants are sufficiently mature and receive the appropriate cues of temperature, day-length and a light quality will they proceed to flower. A greater knowledge of the precise details of these pathways will allow for the selection or breeding of varieties to match particular environments.

## Methods

### Plant material

Three photoperiod responsive wheat cultivars were used for the project: Harnesk and Solstice, both winter varieties, and Paragon, a spring variety. Seeds were planted in 50:50 potting compost:perlite in 10 cm pots and maintained in growth cabinets with a 14 h photoperiod (280) and 16°C day/14°C night temperature. Three weeks post-germination, experimental procedures began (Table 1). As a control, plants of Solstice were grown under identical conditions of light and day-length as the experimental plants but without a corresponding fall in temperature.

Samples were taken for analysis on the last day of each time period/temperature segment: 3, 5, 7, 9 and 12 weeks. Harvesting took place in the middle of the photoperiod to avoid, as much as possible, the influence of circadian rhythms.

### RNA extraction and purification

Total RNA was independently prepared from two separate tissues: crown and leaf. Because of problems of handling, "crown tissue" constituted the apex and some subtending stem including the vascular transition zone and immature leaf bases. In each case, RNA was extracted from material pooled from 4 separate plants. Tissues from control plants were harvested at the same time as the experimental plants. The samples for RNA extraction were frozen in liquid nitrogen, and stored at -80°C until used.

RNA was extracted using the TRIZOL (Invitrogen) method reported in [51].

### Photographic imaging

Crown tissues were collected from plants and fixed and embedded in Lambwax (Raymond A. Lamb Ltd., Eastbourne, BN23 6QE, UK) using standard histochemical techniques [52]. De-parafinised sections were re-hydrated and subsequently stained with 0.05% (w/v) toluidine blue in 10 mM sodium benzoate pH4.4. Slides were examined at low magnification under bright-field illumination using a Wild Photomacroscope IV (Leica Microsystems (UK) Ltd.). A Leica DMRB microscope (Leica Microsystems (UK) Ltd.) was used for high magnification observations and for the capture of photographic images.

### Array analysis

Affymetrix Genechip Wheat Arrays (Affymetrix, USA) were used. Hybridisation, washing and staining were performed according to manufacturer's instructions. Microarray data were extracted from scanned GeneChip images and analyzed using Microarray Suite version 5.0.1 (Affymetrix). Array normalisation and analysis were performed using the GeneSpring GX 7.3 software package (Agilent): the raw data, imported as CEL files, were normalised per chip using RMA (log-scale Robust Multi-array Analysis) open access GeneSpring software; per gene normalisation to the median was performed within the programme. These data are available for public access: the GEO accession number for array data is GSE11774.

### Real-time quantitative RT-PCR

To confirm expression patterns observed from the analysis of array data, real time qRT-PCR experiments were performed using an ABI Prism 7000 SDS (Applied Biosystems, U.K.). Reverse transcription was performed using Superscript III First Strand Synthesis Kits (Invitrogen) according to manufacturer's instructions. Quantitative RT-PCR was performed using SYBR Greener qPCR Supermix for ABI Prism (Invitrogen). The primers used are available in Additional file 6. Reaction conditions were as follows: 50°C for 2 mins; 95°C for 10 mins; 42 cycles of 95°C for 15 s and 58°C 1 min. A dissociation protocol was performed in all cases. Quantification for each primer pair and cDNA template combination was performed in triplicate for each of the two biological replicates at every time point.

Relative quantification was performed using the ΔCt ΔCt method with wheat *ACTIN *used as the endogenous control. Correlation between results from the array data and qRT-PCR was tested using Pearson Correlation Coefficient.

## Authors' contributions

MW carried some of the microarray work, and all of the qRT PCR work. He also performed all of the data analysis and wrote the manuscript. CL grew the plants, collected all tissues for analysis. He performed all RNA extractions and prepared samples for array analysis. JAC performed quality control of RNA and carried out the array analysis. IDW and KJE designed the experimental programme, and gave technical and intellectual guidance.

## Supplementary Material

Additional file 1 **Proposed models for the interaction between the three genes Ta*VRN1*, Ta*VRN2 *and Ta*VRN3***. Models of the interaction between the three genes Ta*VRN1*, Ta*VRN2 *and Ta*VRN3 *(syn. with *TaFT*) redrawn from those proposed by: a) Yan *et al*., 2006; b) Trevaskis *et al*., 2007. Click here for file

Additional file 2 **Vernalised and non-vernalised plants of the wheat varieties Harnesk, Solstice and Paragon**. A) Photographs of non-vernalised plants of varieties Harnesk, Solstice and Paragon 5 months post-germination. B) Photographs of vernalised plants of Harnesk, Solstice and Paragon 5 months post-germination. Click here for file

Additional file 3 **Genes potentially involved in phase transition**. A table listing the features on the Affymetrix Genechip Wheat Genome Array that are reported to be homologues of, or are highly similar to, genes involved in phase transition in *Arabidopsis*. Click here for file

Additional file 4 **Profiles of transcript abundance for photoperiod pathway genes discussed in article**. Profiles of transcript abundance for the photoperiod pathway genes. Only those transcripts that showed a statistically significant 2-fold or greater change in abundance are represented. a) PHYA, b) TOC1, c) LHY, d) GIGANTEA, e) COL1 and f) COL9. Click here for file

Additional file 5 **List of the 42 wheat MADS-box genes described in Zhou *et al*., 2006**. A list of the features on the Affymetrix Genechip Wheat Genome Array that correspond to the wheat MADS-box genes described in the article by Zhou *et al*., 2006 [reference [38]]. Click here for file

Additional file 6 **Primers for qRT-PCR**. A table containing the details of the primer pairs used in qRT-PCR. Click here for file
