# Supplementary material for: Cold- and light-induced changes in the transcriptome of wheat leading to phase transition from vegetative to reproductive growth
Source: BMC Plant Biol. 2009 May 11;9:55. doi: 10.1186/1471-2229-9-55 (PMC2685395; doi:10.1186/1471-2229-9-55)
Supplement: Additional file 1 — Proposed models for the interaction between the three genes TaVRN1, TaVRN2 and TaVRN3. Models of the interaction between the three genes TaVRN1, TaVRN2 and TaVRN3 (syn. with TaFT) redrawn from those proposed by: a) Yan et al., 2006; b) Trevaskis et al., 2007. [file 1471-2229-9-55-S1.doc]

**Additional data in support of manuscript:**

“Cold and light-induced changes in the transcriptome of wheat leading to phase transition from vegetative to reproductive growth”

Mark O. Winfield^1*^, Chungui Lu^2^** Ian D. Wilson^3^, Jane A. Coghill^1^ & Keith J. Edwards^1^

**Proposed models for the interaction between the three genes Ta*VRN1*, Ta*VRN2* and Ta*VRN3***

a) is redrawn from Yan *et al.*, 2006; b) is redrawn form Trevaskis *et al.*, 2007. The red boxes indicate that the gene tends to retard flowering, the green boxes indicate that the gene tends to promote flowering. LD = long days; SD = short days.


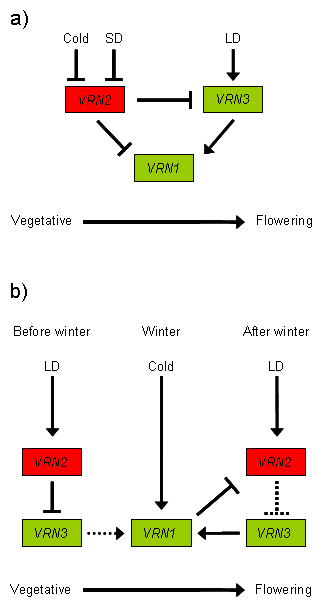


**References**

B Trevaskis, MN Hemming, ES Dennis, WJ Peacock: **The molecular basis of vernalisation-induced flowering in cereals.** *Trends in Plant Science* 2007, **12**:352-357.

L Yan, D Fu, C Li, A Blechl, G Tranquilli, M Bonafede, A Sanchez, M Valarik, S Yasuda, J Dubcovsky: **The wheat and barley vernalization gene VRN3 is an orthologue of FT**. *Proceedings of the National Academy of Sciences of the United States of America* 2006, **103**:19581-19586.
