# Supplementary material for: Cold- and light-induced changes in the transcriptome of wheat leading to phase transition from vegetative to reproductive growth
Source: BMC Plant Biol. 2009 May 11;9:55. doi: 10.1186/1471-2229-9-55 (PMC2685395; doi:10.1186/1471-2229-9-55)
Supplement: Additional file 2 — Vernalised and non-vernalised plants of the wheat varieties Harnesk, Solstice and Paragon. A) Photographs of non-vernalised plants of varieties Harnesk, Solstice and Paragon 5 months post-germination. B) Photographs of vernalised plants of Harnesk, Solstice and Paragon 5 months post-germination. [file 1471-2229-9-55-S2.doc]

**Additional data in support of manuscript:**

“Cold and light-induced changes in the transcriptome of wheat leading to phase transition from vegetative to reproductive growth”

Mark O. Winfield^1*^, Chungui Lu^2^** Ian D. Wilson^3^, Jane A. Coghill^1^ & Keith J. Edwards^1^

**Vernalised and non-vernalised plants of the wheat varieties Harnesk, Solstice and Paragon**

**A.** Non-vernalised plants 5 months post-germination: a) Harnesk; b) Solstice; c) Paragon. The two winter varieties, Harnesk and Solstice, show no sign of stem elongation, booting or flowering. The spring variety, Paragon, has headed fully and is at anthesis.


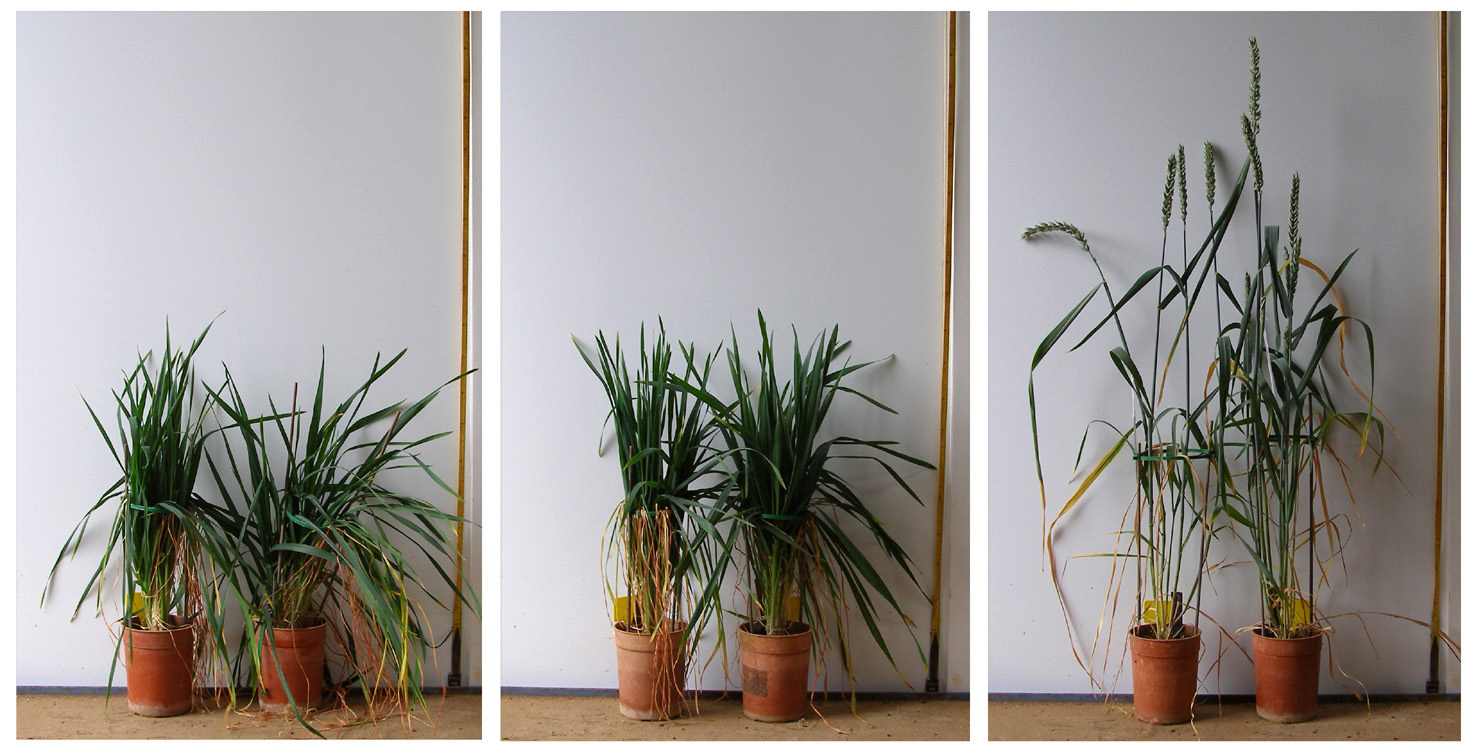


a)

b)

c)

**B.** Vernalised plants 5 months post-germination**:** a) Harnesk; b) Solstice; c) Paragon. Plants of Harnesk have booted fully and are just showing signs of heading. Plants of both Solstice and Paragon have headed fully.


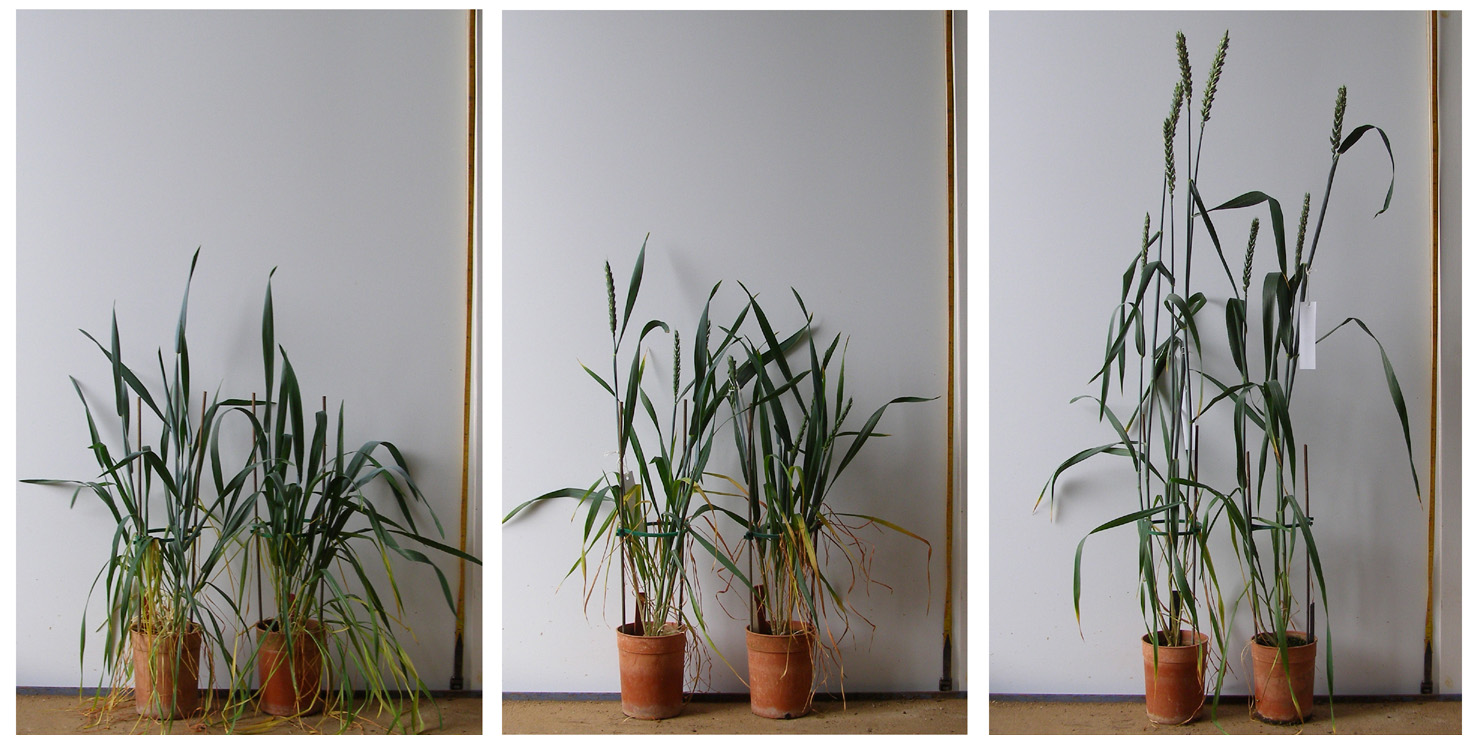


a)

b)

c)
