# Supplementary material for: Cold- and light-induced changes in the transcriptome of wheat leading to phase transition from vegetative to reproductive growth
Source: BMC Plant Biol. 2009 May 11;9:55. doi: 10.1186/1471-2229-9-55 (PMC2685395; doi:10.1186/1471-2229-9-55)
Supplement: Additional file 3 — Genes potentially involved in phase transition. A table listing the features on the Affymetrix Genechip Wheat Genome Array that are reported to be homologues of, or are highly similar to, genes involved in phase transition in Arabidopsis. [file 1471-2229-9-55-S3.doc]

**Additional data in support of manuscript:**

“Cold and light-induced changes in the transcriptome of wheat leading to phase transition from vegetative to reproductive growth”

Mark O. Winfield^1*^, Chungui Lu^2^** Ian D. Wilson^3^, Jane A. Coghill^1^ & Keith J. Edwards^1^

**Genes potentially involved in phase transition**

| *Arabidopsis genes* | | | Probe-set(s) on wheat genome array |
| --- | --- | --- | --- |
|  |  |  |  |
|  | Gibberellin Pathway | |  |
|  |  | *GA1 (GA REQUIRING 1*) - *ent*-copalyl diphosphate synthase | NO MATCH |
|  |  | *GA2 - ent*-kaurene synthase | Ta.8418.1, TaAffx.80963.1 |
|  |  | *GA3 - ent*-kaurene oxidase | Ta.14904.1 |
|  |  | *GAI* (*GA INSENSITIVE*) | NO MATCH |
|  |  | *RGA1* (*REPRESSOR OF GA*) | TaAffx.143995.10.S1_x_at |
|  |  |  |  |
|  | Autonomous Pathway | |  |
|  |  | *FLD* | NO MATCH |
|  |  | *FVE* | Ta.3385.1 |
|  |  | *FPA* | NO MATCH |
|  |  | *FCA* | Ta.5855.2.S1_x_at, TaAffx.98485.9, 5, 6, 10. |
|  |  | *FY* | NO MATCH |
|  |  | *LD* (*LUMIDEPENDENS*) | TaAffx.37264.1.S1_at |
|  |  | *FRI* (*FRIGIDA*) | NO ORTHOLOGUE IN MONOCOTS (Yan *et al.*, 2003) |
|  |  |  |  |
|  | Vernalisation Pathway | |  |
|  |  | *VRN1* (*VERNALISATION RESPONSE 1*) | Ta.9525.1.S1_x_at |
|  |  | *VRN2* (*VERNALISATION RESPONSE 2*) | Ta.367.1.S1_at = *EMBRYONIC FLOWER 2* |
|  |  | *VIN3* (*VERNALISATION INSENSITIVE 3*) | Ta.10289.2 = *TmVIL1* |
|  |  |  | Ta.7238.1 and TaAffx.92348.1 = *TmVIL2* |
|  |  |  | Ta.16935.1 = *TmVIL3* |
|  |  | *VERNALISATION INDEPENDENT 4 VIP4* | Ta.6967.2.S1_at |
|  |  | *FLC* (*FLOWERING LOCUS C*) | NO ORTHOLOGUE IN MONOCOTS (Yan *et al.*, 2003) |
|  |  |  |  |
|  | Photoperiod Pathway | |  |
|  |  | *CRY1* (CRYPTOCHROME *1*) | TaAffx.49997.1.S1_at |
|  |  | *CRY2* (*CRYPTOCHROME 2*) | TaAffx.49997.1.S1_at |
|  |  | *PHYA* (*PHYTOCHROME A*) | Ta.7896.1 |
|  |  | *PHYB* (*PHYTOCHROME B*) | NO MATCH |
|  |  | *PHYC* (*PHYTOCHROME C*) | TaAffx.116496.1.S1_at |
|  |  | *PHYD* (*PHYTOCHROME D*) | NO MATCH |
|  |  | *PHYE* (*PHYTOCHROME E*) | NO MATCH |
|  |  | *LHY* (*LATE ELONGATED HYPOCOTYL*) | Ta.27013.1.S1_at |
|  |  | *CCA1* (*CIRCARDIAN CLOCK ASSOCIATED 1*) | Ta.7524.1.A1_at This is LHY |
|  |  | *ELF3* (*EARLY FLOWERING 3*) | TaAffx.54485.1.S1_at, TaAffx.71213 |
|  |  | *TOC1* | Ta.12422 |
|  |  | *ESD4* | Ta.27700.1.S1_at |
|  |  | *GI* (*GIGANTEA*) | Ta.10215.1 |
|  |  | *CO* (*CONSTANS*) | Ta.28785.1.S1_at |
|  |  |  |  |
|  | Intergrative Pathway | |  |
|  |  | *FD* | Ta.30801.1.S1_at |
|  |  | *FWA* | Ta.6436.1.S1_at |
|  |  | *PDF2* | Ta.1998.2.S1_a_at |
|  |  | *FT* (*FLOWERING LOCUS T*) | Ta.30640.1.A1_at |
|  |  | SOC1 (*SUPRESSOR OF OVEREXPRESSION OF CO1*) | Ta.19661 |
|  |  |  |  |
|  | Meristem Identity Genes | |  |
|  |  | *LFY* (*LEAFY*) | NO MATCH |
|  |  | *TFL1* (*TERMINAL FLOWER 1*) | TaAffx.36760.1.S1_at |
|  |  | *APETALA 1 AP1* | TaAffx.143995.17.A1_at; Ta.142.1.S1_at |

Features on the AFFYMETRIX GENECHIP WHEAT GENOME ARRAY that are reported as homologues of, or are highly similar to, genes involved in phase transition in *Arabidopsis*.
