# Supplementary material for: Cold- and light-induced changes in the transcriptome of wheat leading to phase transition from vegetative to reproductive growth
Source: BMC Plant Biol. 2009 May 11;9:55. doi: 10.1186/1471-2229-9-55 (PMC2685395; doi:10.1186/1471-2229-9-55)
Supplement: Additional file 4 — Profiles of transcript abundance for photoperiod pathway genes discussed in article. Profiles of transcript abundance for the photoperiod pathway genes. Only those transcripts that showed a statistically significant 2-fold or greater change in abundance are represented. a) PHYA, b) TOC1, c) LHY, d) GIGANTEA, e) COL1 and f) COL9. [file 1471-2229-9-55-S4.doc]

**Additional data in support of manuscript:**

“Cold and light-induced changes in the transcriptome of wheat leading to phase transition from vegetative to reproductive growth”

Mark O. Winfield^1*^, Chungui Lu^2^** Ian D. Wilson^3^, Jane A. Coghill^1^ & Keith J. Edwards^1^

**Profiles of transcript abundance for photoperiod pathway genes discussed in article**

Profiles of transcript abundance (linear scale) for genes discussed in article: a) PHYA, b) TOC1, c) LHY, d) GIGANTEA, e) COL1 and f) COL9. The X axis scale is weeks post-germination. The regime of declining temperature and light began at 3 weeks (average day/night temperature to three weeks was 15 ^o^C with day-length of 14 hours). Average day/night temperature at 5 weeks was 12 ^o^C with a day-length of 12 hours; average day/night temperature at 9 weeks = 6 ^o^C with a day-length of 9 hours). Harnesk and Solstice are winter varieties of wheat, Paragon a spring variety. Only those transcripts that showed a statistically significant 2-fold or greater change in abundance are represented.

**a)**


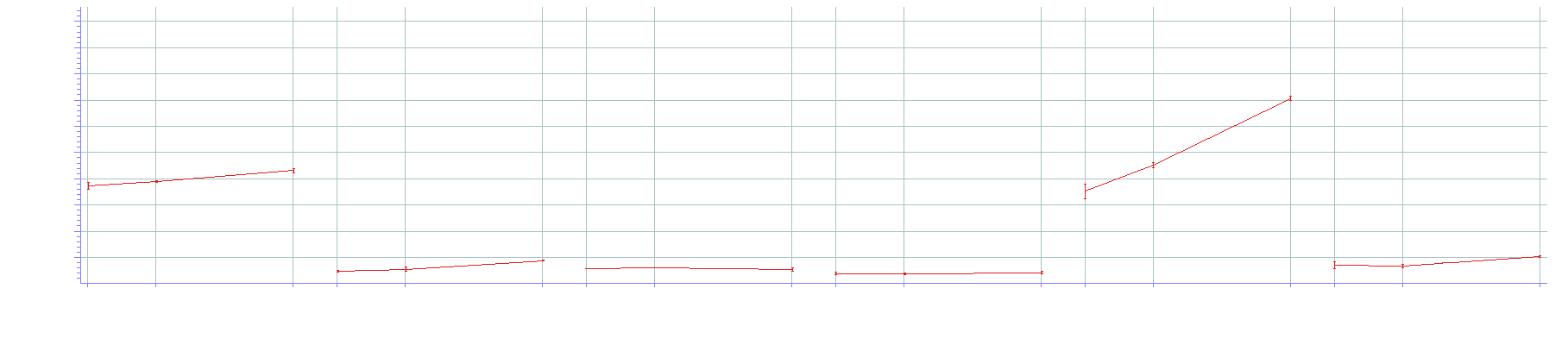


3

5

9

3

5

9

3

5

9

3

5

9

3

5

9

3

5

9

Harnesk

Crown

Harnesk

Leaf

Paragon

Crown

Paragon

Leaf

Solstice

Crown

Solstice

Leaf

0

2

4

6

8

10

PHYA

**b)**


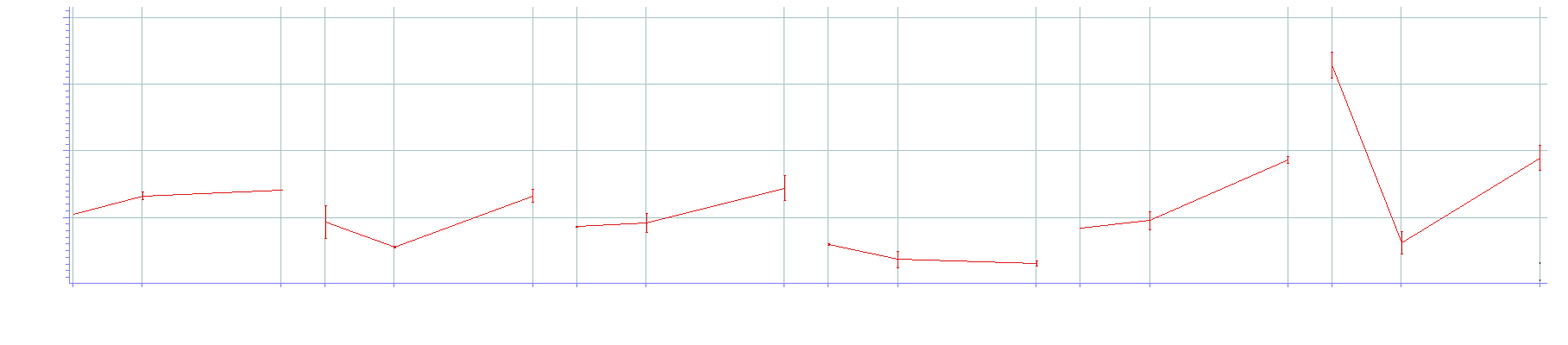


3

5

9

3

5

9

3

5

9

3

5

9

3

5

9

3

5

9

Harnesk

Crown

Harnesk

Leaf

Paragon

Crown

Paragon

Leaf

Solstice

Crown

Solstice

Leaf

0

1

2

3

4

TOC1

**c)**


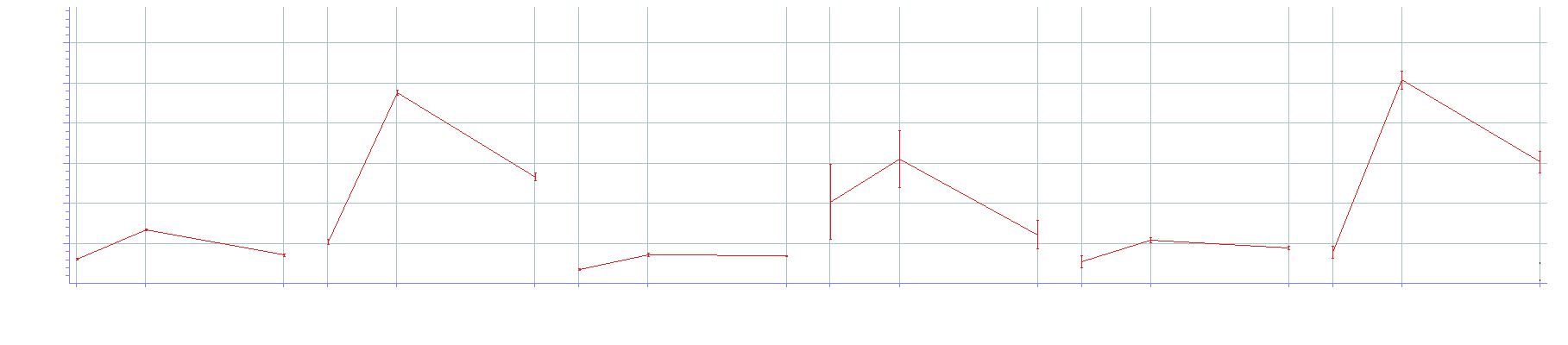


3

5

9

3

5

9

3

5

9

3

5

9

3

5

9

3

5

9

Harnesk

Crown

Harnesk

Leaf

Paragon

Crown

Paragon

Leaf

Solstice

Crown

Solstice

Leaf

0

2

4

6

LHY

**d)**


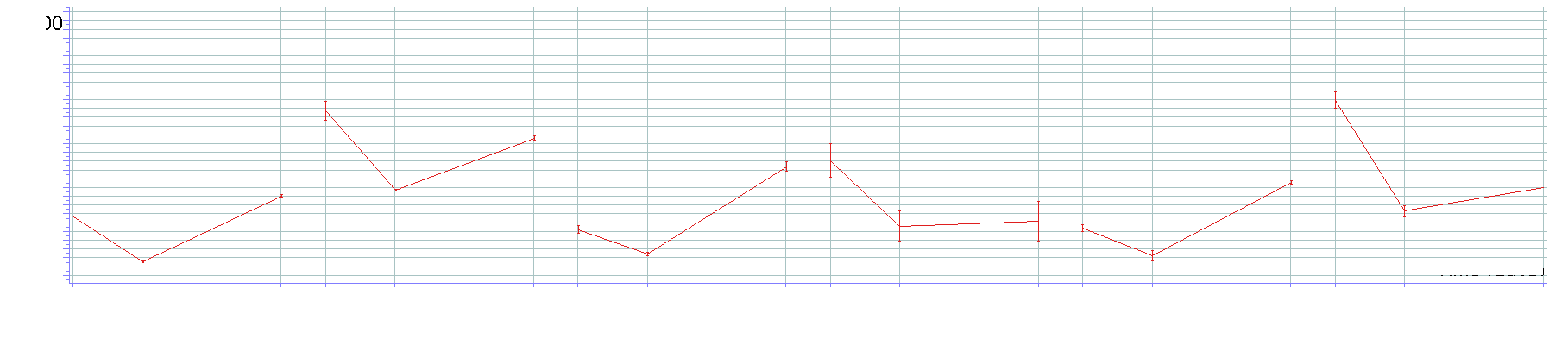


3

5

9

3

5

9

3

5

9

3

5

9

3

5

9

3

5

9

Harnesk

Crown

Harnesk

Leaf

Paragon

Crown

Paragon

Leaf

Solstice

Crown

Solstice

Leaf

0.0

0.5

1.0

1.5

2.0

2.5

GIGANTEA

**e)**


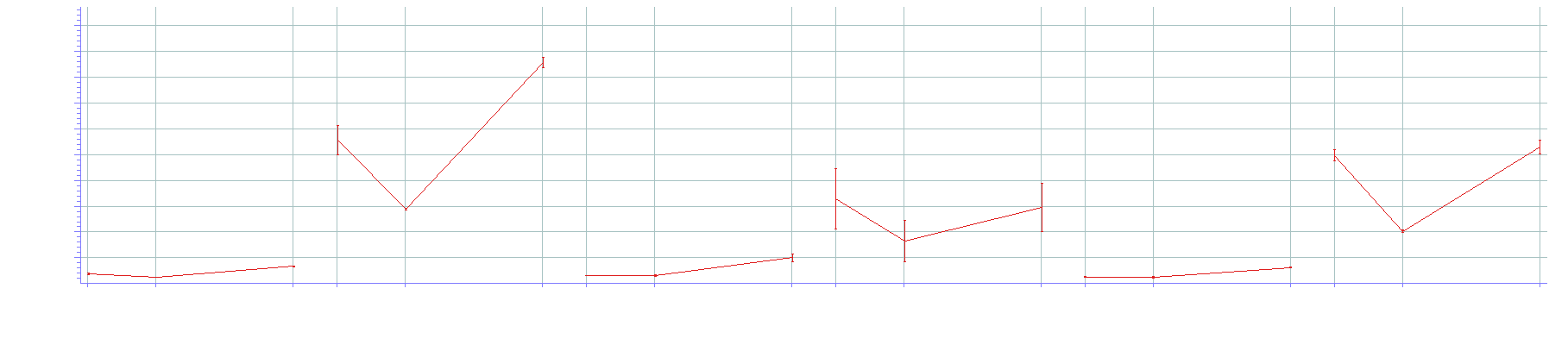


3

5

9

3

5

9

3

5

9

3

5

9

3

5

9

3

5

9

Harnesk

Crown

Harnesk

Leaf

Paragon

Crown

Paragon

Leaf

Solstice

Crown

Solstice

Leaf

0

2

4

6

8

10

COL1

**f)**


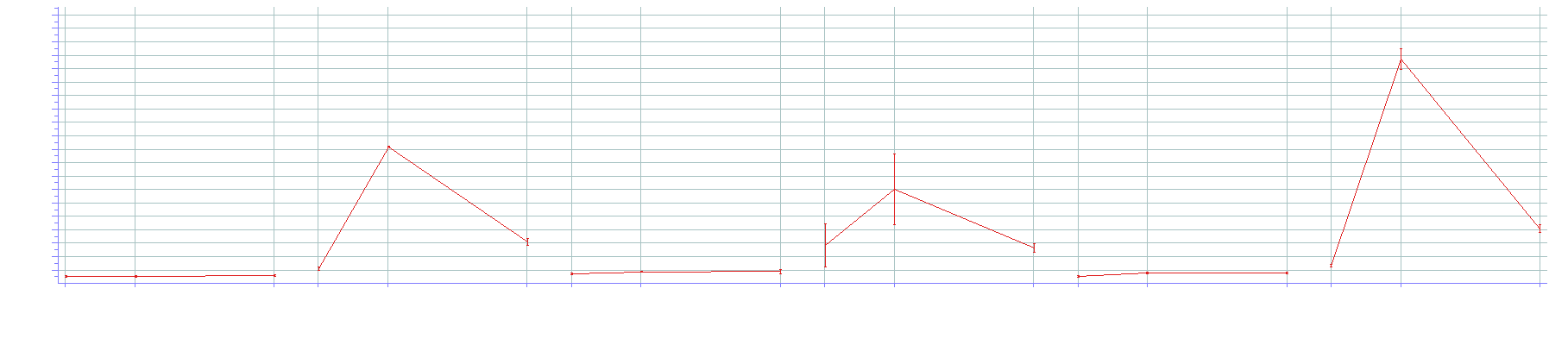


0

5

10

20

15

COL9

3

5

9

3

5

9

3

5

9

3

5

9

3

5

9

3

5

9

Harnesk

Crown

Harnesk

Leaf

Paragon

Crown

Paragon

Leaf

Solstice

Crown

Solstice

Leaf
