# Supplementary material for: Cold- and light-induced changes in the transcriptome of wheat leading to phase transition from vegetative to reproductive growth
Source: BMC Plant Biol. 2009 May 11;9:55. doi: 10.1186/1471-2229-9-55 (PMC2685395; doi:10.1186/1471-2229-9-55)
Supplement: Additional file 5 — List of the 42 wheat MADS-box genes described in Zhou et al., 2006. A list of the features on the Affymetrix Genechip Wheat Genome Array that correspond to the wheat MADS-box genes described in the article by Zhou et al., 2006 [reference [38]]. [file 1471-2229-9-55-S5.doc]

**Additional data in support of manuscript:**

“Cold and light-induced changes in the transcriptome of wheat leading to phase transition from vegetative to reproductive growth”

Mark O. Winfield^1*^, Chungui Lu^2^** Ian D. Wilson^3^, Jane A. Coghill^1^ & Keith J. Edwards^1^

**Table A2.** List of the 42 wheat MADS-box genes described in Zhou *et al.*, 2006.

| Gene Name | NCBI  Unique Identifier | Probe-set(s) on wheat genome array |
| --- | --- | --- |
|  |  |  |
| TaAGL6 | DQ512329 | NO MATCH |
| TaAGL12 | DQ512367 | NO MATCH |
| TaAGL39 | DQ512355 | Ta.12700.1 |
| TaAGL16 | DQ512334 | Ta.14469.3 |
| TaAGL28 | DQ512345 | Ta.14469.3 |
| TaAGL30 | DQ512348 | Ta.14469.3 |
| TaAGL1 | DQ512330 | Ta.21250.1 |
| TaAGL18 | DQ512335 | Ta.21250.1 |
| TaAGL23 | DQ512340 | Ta.21250.1 |
| TaAGL22 | DQ512365 | Ta.22382.1 |
| TaAGL7 | DQ512363 | Ta.25343.1 |
| TaAGL32 | DQ512350 | Ta.26215.1 |
| TaAGL41 | DQ512357 | Ta.26917.1 |
| TaAGL35 | DQ512369 | Ta.27569.1 |
| TaAGL17 | DQ512368 | Ta.27569.1 |
| TaAGL2 | DQ512337 | Ta.27776.1 |
| TaAGL9 | DQ512364 | Ta.27776.1 |
| TaAGL31 | DQ512349 | Ta.27776.1 |
| TaAGL25* | DQ512342 | Ta.30607.1, Ta.142, TaAffx.143995.17 |
| TaAGL3 | DQ512347 | Ta.3395.1 |
| TaAGL5 | DQ512362 | Ta.3395.1 |
| TaAGL8 | DQ512359 | Ta.3395.1 |
| TaAGL34 | DQ512351 | Ta.3395.1 |
| TaAGL40 | DQ512370 | Ta.3395.1 |
| TaAGL29 | DQ512346 | Ta.3583.1 |
| TaAGL4 | DQ512356 | Ta.3854.1 |
| TaAGL24 | DQ512341 | Ta.3854.1 |
| TaAGL26 | DQ512343 | Ta.4889.1 |
| TaAGL14 | DQ512360 | Ta.6411.1 |
| TaAGL15 | DQ512361 | Ta.6411.1 |
| TaAGL11 | DQ512332 | Ta.6743.1 |
| TaAGL13 | DQ512333 | Ta.7339.1 |
| TaAGL19 | DQ512336 | Ta.7339.1 |
| TaAGL37 | DQ512353 | Ta.777.1 |
| TaAGL36** | DQ512352 | TaAffx.10868.1 |
| TaAGL10 | DQ512331 | TaAffx.120063.1 |
| TaAGL27 | DQ512344 | TaAffx.143995.19 |
| TaAGL20 | DQ512338 | TaAffx.19661.1 |
| TaAGL21 | DQ512339 | TaAffx.19661.1 |
| TaAGL38 | DQ512354 | TaAffx.19661.1 |
| TaAGL42 | DQ512358 | TaAffx.65068.1 |
| TaAGL33 | DQ512366 | TaAffx.6606.1, Ta.26917.1 |

Genes have been grouped according to their AFFYMETRIX GENECHIP WHEAT GENOME ARRAY probe-set identifier. *Synonym of VRN1; ** synonym of VRT-2. Where several MADS-box genes correspond to the same probe-set on the array, they are highlighted by blocks of colour.

**Reference**

T Zhao, ZF Ni, Y Dai, YY Yao, XL Nie, QX Sun**: Characterization and expression of 42 MADS-box genes in wheat (Triticum aestivum L**.)*. Molecular Genetics and Genomic*s 2006**, 2**76:334-350.
