# Supplementary material for: Cold- and light-induced changes in the transcriptome of wheat leading to phase transition from vegetative to reproductive growth
Source: BMC Plant Biol. 2009 May 11;9:55. doi: 10.1186/1471-2229-9-55 (PMC2685395; doi:10.1186/1471-2229-9-55)
Supplement: Additional file 6 — Primers for qRT-PCR. A table containing the details of the primer pairs used in qRT-PCR. [file 1471-2229-9-55-S6.doc]

**Additional data in support of manuscript:**

“Cold and light-induced changes in the transcriptome of wheat leading to phase transition from vegetative to reproductive growth”

Mark O. Winfield^1*^, Chungui Lu^2^** Ian D. Wilson^3^, Jane A. Coghill^1^ & Keith J. Edwards^1^

**Primers for qRT-PCR**

| Oligo_Name | Sequence | Product Length | Pearson  Correlation with array data |
| --- | --- | --- | --- |
| AGL10-F | CTCAAGAAGGCACACGAGAT | 203 | 0.43 |
| AGL10-R | TATTCATCTCCCCAGTTTGC |  |  |
|  |  |  |  |
| AGL33-F | AACCCGGTGCAAGTAACAAC | 207 | 0.82 |
| AGL33-R | CACTCGTTCCTGCACCACTA |  |  |
|  |  |  |  |
| AGL42-F | GGCTTTGGCGAAACAAAATA | 200 | 0.78 |
| AGL42-R | TAGCAGCCCTGGCAACTAAT |  |  |
|  |  |  |  |
| COL9-F | CAATTAACCCGACCGAAAAA | 187 | 0.55 |
| COL9-R | CGCTCGTAGCAAGCAGACAT |  |  |
|  |  |  |  |
| ENT KO-F | CATTCTCCTGTCCCACTGGT | 194 | 0.98 |
| ENT KO-R | GAGCCGAATGACATGGTTTT |  |  |
|  |  |  |  |
| VRN1-F | GCGCAACAAGATCAGACTCA | 219 | 0.76 |
| VRN1-R | ACGCTTATATGGGCTGGAAG |  |  |
|  |  |  |  |
| VRN2-F | TTCCGGTAATTTATAGCACAAGC | 122 | 0.95 |
| VRN2-R | GGCTCCAATCGATCAATCAC |  |  |
|  |  |  |  |
| Actin-F | GATACACGCTTCCTCATGCT | 181 |  |
| Actin-R | TCCAGCTCCTGCTCATAATC |  |  |
|  |  |  |  |

All primer pairs used were designed to work at an annealing temperature of 58°C.
